# Thermally Targeted Delivery of a c-Myc Inhibitory Polypeptide Inhibits Tumor Progression and Extends Survival in a Rat Glioma Model

**DOI:** 10.1371/journal.pone.0055104

**Published:** 2013-01-25

**Authors:** Gene L. Bidwell, Eddie Perkins, Joshua Hughes, Majid Khan, Judy R. James, Drazen Raucher

**Affiliations:** 1 Department of Neurology, University of Mississippi Medical Center, Jackson, Mississippi, United States of America; 2 Cancer Institute, University of Mississippi Medical Center, Jackson, Mississippi, United States of America; 3 Department of Neurosurgery, University of Mississippi Medical Center, Jackson, Mississippi, United States of America; 4 Department of Neurobiology and Anatomical Sciences, University of Mississippi Medical Center, Jackson, Mississippi, United States of America; 5 Department of Radiology, University of Mississippi Medical Center, Jackson, Mississippi, United States of America; 6 Department of Biochemistry, University of Mississippi Medical Center, Jackson, Mississippi, United States of America; City of Hope National Medical Center and Beckman Research Institute, United States of America

## Abstract

Treatment of glioblastoma is complicated by the tumors’ high resistance to chemotherapy, poor penetration of drugs across the blood brain barrier, and damaging effects of chemotherapy and radiation to normal neural tissue. To overcome these limitations, a thermally responsive polypeptide was developed for targeted delivery of therapeutic peptides to brain tumors using focused hyperthermia. The peptide carrier is based on elastin-like polypeptide (ELP), which is a thermally responsive biopolymer that forms aggregates above a characteristic transition temperature. ELP was modified with cell penetrating peptides (CPPs) to enhance delivery to brain tumors and mediate uptake across the tumor cells’ plasma membranes and with a peptide inhibitor of c-Myc (H1). In rats with intracerebral gliomas, brain tumor targeting of ELP following systemic administration was enhanced up to 5-fold by the use of CPPs. When the lead CPP-ELP-fused c-Myc inhibitor was combined with focused hyperthermia of the tumors, an additional 3 fold increase in tumor polypeptide levels was observed, and 80% reduction in tumor volume, delayed onset of tumor-associated neurological deficits, and at least doubled median survival time including complete regression in 80% of animals was achieved. This work demonstrates that a c-Myc inhibitory peptide can be effectively delivered to brain tumors.

## Introduction

Glioblastoma multiforme (GBM) is the most common and aggressive form of malignant brain tumor [1]. Treatment for GBM involves surgical removal of as much tumor as possible, followed by radiation therapy and/or chemotherapy with the alkylating agent temozolomide [2]. The anti-angiogenic monoclonal antibody bevacizumab has also been approved for GBM therapy for refractory tumors. However, even with aggressive therapy, the median survival of patients with GBM is only 12–24 months [3]. Treatment of GBM is complicated by several factors. The tumors are highly resistant to chemotherapeutics, and the blood brain barrier (BBB) makes delivery of therapeutic agents to GBM tumors exceedingly difficult [2]. Also, the susceptibility of nonmalignant neural tissue to chemotherapy and radiotherapy damage, and its inability to easily repair itself, further complicate the development of novel treatments for GBM. Given the ineffectiveness of current treatment options, there is a critical need to develop therapeutic strategies that can deliver agents to GBM tumors effectively, inhibit proliferation of tumor cells potently, and spare adjacent non-malignant neural tissue, thereby reducing treatment-related side effects.

Peptide therapeutics are a novel class of agents for cancer therapy. Therapeutic peptides (TPs) are capable of modulating important protein/protein interactions and eliciting a therapeutic response. There are many examples of TPs targeted to known oncogenes [4], [5], and, if used in the right context in which the TP is matched to a tumor-specific oncogenic lesion, TPs have great promise as targeted and personalized agents. The advantages of TPs lie in their ease of design for any target protein and in their specificity for that target. However, their use is limited by their poor stability and inefficient ability to penetrate biological membranes [6], [7]. In order to overcome these limitations and make peptides viable biopharmaceuticals, a suitable carrier system is needed that can stabilize the cargo peptide in circulation, target the peptide to the desired tumor site, and facilitate the penetration of the peptide into the tumor cell and to the intracellular site of action [8].

We have developed a polypeptide-based carrier for TPs that is capable of delivering the peptide cargo across the plasma membrane of the target cell and directing its intracellular localization [4], [9]. This carrier, based on elastin-like polypeptide (ELP), is a thermally responsive biopolymer that reversibly forms aggregates at a pre-defined transition temperature (T_t_) [10]. The thermally responsive property of ELP can be exploited to direct its accumulation *in vivo* to the site of externally applied, focused, mild hyperthermia, a process known as thermal targeting [11]–[13]. ELP was modified with a cell penetrating peptide (CPP), which mediates its cellular uptake and subcellular distribution, and with a TP targeted to the oncogenic protein c-Myc. This TP is derived from helix 1 (H1) of the helix-loop-helix domain of c-Myc, and it functions by blocking the endogenous c-Myc/Max interaction and preventing activation of transcription by c-Myc and Max [14]. This polypeptide has antiproliferative effects in breast, cervical, and uterine cancer cell lines [15], [16]. It has been shown that 78% of human GBM tumors express c-Myc [17], and c-Myc expression level has been correlated with tumor grade [18]. Although c-Myc gene amplification or rearrangement is uncommon in GBM [19], concomitant inactivation of p53 and PTEN was found to be a common event in a subset of primary GBMs, and simultaneous inactivation of p53 and PTEN lead to increased c-Myc expression and an undifferentiated phenotype in murine neural stem cells [20]. Furthermore, glioma cancer stem cells have been shown to express c-Myc at much higher levels than non-stem glioma cells, and c-Myc knockdown was shown to reduce proliferation and cause cell cycle arrest in glioma stem cells [21]. Based on these results, we hypothesized that our ELP-fused c-Myc inhibitory peptide (CPP-ELP-H1) might be efficacious for inhibition of glioma progression.

In this study, we used a rat model of glioma generated by intracranial implantation of C6 glioma cells to determine the pharmacokinetics, tumor targeting, and tumor inhibition of the CPP-ELP-delivered H1 peptide. The C6 model was chosen because it closely resembles the human disease both histologically and in its aggressive nature [22] (including developing a rim of reactive astrocytes, a highly vascularized and BrdU positive tumor periphery, and a necrotic core [22], [23]), and because the use of immunocompetent Sprague Dawley rats allows better assessment of toxicity and potential immunogenicity of the test agents than do nude mouse models. Also, c-Myc expression has been demonstrated in both cultured C6 cells and orthotopic C6 xenografts [24], [25]. We confirmed that the orthotopic C6 tumors used here consisted of densely packed tumor cells, were highly vascularized, did not express GFAP, and were surrounded by GFAP positive astrocytes (Figure S1), all consistent with previous reports [22], [23].

## Materials and Methods

### Synthesis, Purification, and Labeling of Polypeptides

The Bac-ELP-H1 polypeptide has the amino acid sequence MRRIRPRPPRLPRPRPRPLPFPRPGGCYPG-(VPGXG)n-WPGSGNELKRAFAALRDQI. Bac-ELP1-H1 contains V,G, or A at the X position in a 5∶3:2 ratio, respectively, and n is 150. Bac-ELP2-H1 contains V,G, or A at the X position in a 1∶7:8 ratio, respectively, and n is 160. ELP polypeptides were expressed in E. coli and purified by thermal cycling as described in [15], and labeled on a unique cysteine residue with tetramethylrhodamine-5-maleimide or AlexaFluor® 750 C_5_-maleimide (Invitrogen) [15]. Excess unreacted label was removed by thermal cycling, and extent of labeling was determined spectrophotometrically.

### Cell Culture and Polypeptide Treatment

Human U-87-MG and D54 glioma cells along with the rat C6 glioma cells were obtained from American Type Culture Collection (ATCC). Cells were maintained in Dulbecco’s Modified Eagle Medium (DMEM) supplemented with 10% fetal bovine serum (Atlanta Biologicals), 100 U/mL penicillin, 100 µg/mL streptomycin, and 25 µg/mL amphotericin B (Invitrogen) at 37°C and 5% CO_2_. For proliferation assays, cells were seeded in 6-well plates (25,000 cells/mL for U-87-MG or D54 cells, 5,000 cells/mL for C6 cells). The following day, cells were treated with the indicated concentration of CPP-ELP1-H1 for 1 h at 37°C or 42°C, the polypeptide was removed and replaced with fresh medium, and cells were allowed to proliferate for 72 h under normal cell culture conditions. Cell number was determined using the MTS cell proliferation assay (Promega). The data shown represent the mean ± s.e. of three independent experiments.

### Tumor Implantation

All animal manipulations were performed in accordance with the National Institutes of Health Guide for the Care and Use of Laboratory Animals and were approved by the University of Mississippi Medical Center’s Institutional Animal Care and Use Committee. All surgery was performed under isoflurane anesthesia, and all efforts were made to minimize suffering. C6 cells were harvested from 50%–75% confluent flasks, rinsed, and resuspended in PBS at a concentration of 40,000 cells/mL. Female Sprague Dawley rats (Charles River) were anesthetized with isoflurane, and the head was shaved and prepared for surgery with Povidone iodine. A midline scalp incision was made and a small hole was bored on the left side of the skull, 2 mm posterior to the Bregma and 2 mm lateral to the sagittal suture. 200,000 cells in a volume of 5 µL were drawn into a Hamilton syringe (Hamilton Co., Reno NV) fitted with a blunt 23 gauge needle and a depth stop at 4 mm. The cell volume was injected in the intra striatal region. The syringe was slowly retracted, the hole was sealed with bone wax, the incision was flooded with Sensorcaine (0.5%), and the skin was closed with 5–0 Vicryl suture. Acute biodistribution experiments were performed 10–12 days after tumor implantation. For tumor reduction studies, 8 days after implantation, tumor formation was confirmed by MRI scanning as described below.

### Pharmacokinetics

10 to 12 days after tumor implantation, rats were anesthetized with isoflurane, and a cannula was placed in the femoral artery. Rhodamine-labeled CPP-ELP1 or ELP1 (100 mg/kg) was administered by IV injection into the femoral vein. 30 µL of blood was sampled at the indicated time points over a period of 4 h using the arterial catheter. Blood was collected in heparinized hematocrit tubes and centrifuged (13,000×g, 5 min) to separate the plasma from the cells. 1 min prior to euthanasia, 500 kDa FITC-dextran (20 mg/kg, Sigma) was injected IV to mark the perfused vasculature. Polypeptide fluorescence in the plasma was determined using a fluorescence plate reader (Bio Tek), and a standard curve was generated using known quantities of the injected polypeptide. Fluorescence data were fit to the standard curve to calculate plasma levels (µg/mL) at each sampling point. Plasma clearance data were fit to a two-compartment pharmacokinetic model using Microcal Origin as described in [13].

### Quantitative Fluorescence Analysis

Acute biodistribution studies were performed using organs harvested 4 h after the administration described above. Major organs were harvested at necropsy, rapidly frozen, cut into 15 µm sections using a cryo-microtome, and mounted onto slides. Fluorescence standards were made by freezing known quantities of the injected protein and cutting discs to the same thickness as tissue sections. Standards were made from the same batch of polypeptide used for the animal injections, which allows for correction for any variation in labeling efficiency from one polypeptide to the next. Tissue sections and standards were scanned using a ScanArray Express slide scanner (Perkin Elmer). Fluorescence intensity of sections and standards was determined using Image J software, and the amount of injected protein/gram of tissue was calculated by fitting the tumor or organ fluorescence intensity to the standard curve after subtraction of autofluorescence from control, saline injected organs. Similar methods were used to determine tumor levels versus plasma levels in each animal at 5 min and at 4 h after injection and to determine tumor levels versus contralateral normal brain and versus CSF in ventricles. Tumor and organ levels were averaged for all animals (n = 6 rats/group), and data represent the mean ± s.e. Microscopic images of tumor sections were collected using a Nikon epifluoresence microscope with a 60× objective and MetaMorph software.

### Tumor Heating Using Infrared Light

For hyperthermia experiments, rats were anesthetized with isoflurane, the surgical area was prepared as above, and the scalp incision was re-opened. A small craniotomy was performed over the tumor site using a drill fitted with a small burr. The skull over laying the injection site (1 cm diameter) was drilled away exposing the dura mater. Tumor heating was performed by illumination of the tumor using the SLD/LED cluster of a Laser Sys-Stim 540® (Mettler Electronics). The tumor was heated by continuous illumination for 20 min, followed by a 10 min cooling period, and this protocol was repeated for 4 cycles. The area surrounding the tumor site was shielded from illumination. For determining the extent of tumor hyperthermia, the tumor core temperature was measured using a 30 ga needle thermocouple connected to a HHM290 Supermeter (Omega Engineering). Body temperature was monitored with a rectal probe and maintained at 37°C using a homeothermic blanket (Harvard Apparatus). Following the heating protocol, the incision was flooded with Sensorcaine (0.5%) and sutured with 5–0 Vicryl. The data shown represent the mean of 3 animals.

### *Ex Vivo* Fluorescence Imaging

To determine the effect of thermal targeting, polypeptide levels in tumors and major organs were determined after IV administration of AlexaFluor750-labeled Bac-ELP1-H1 or AlexaFluor750-labeled Bac-ELP2-H1 (100 mg/kg) by *ex vivo* fluorescence whole-organ imaging with an IVIS Spectrum (Caliper). 10 to 12 days after tumor implantation, polypeptides or saline control were injected IV via the femoral vein and, in heated groups, the tumor was heated using the thermal cycling protocol described above. 4 h after injection, the rats were sacrificed and the brain and other organs were removed. Tumor and organ fluorescence was quantified using Living Image software (Caliper). The tumor and organ fluorescence was averaged for all animals (n = 4 rats/group), and the data represent the mean ± s.e.

### Monitoring Tumor Progression by MRI Scanning

Tumor volumes were determined by MRI scanning. Rats were anesthetized with an IP injection of ketamine (60 mg/kg) and xylazine (7 mg/kg) and administered a 0.1 cc dose of OptiMark contrast agent in 1 cc of saline IP. *In vivo* MRI data of the intracranial C6 tumors was collected on a 70 cm bore 1.5 T Siemens ESPREE MRI scanner (Erlangen, Germany). The magnet was equipped with 76 seamlessly integrated coil elements with up to 18 RF channels. Imaging was done at the iso-center of the magnet using a radiofrequency (RF) phased-array dedicated wrist coil tuned to proton frequency. Two rats were placed in the coil with the rat brains aligned with the center of the coil. Multi-slice 3D T1 Volumetric-Interpolated-Breath-Hold Examination (VIBE) trans-axial images were acquired to evaluate the structural changes of the tumors with a repetition time (TR) of 20 ms, echo time (TE) of 7.15 ms and data matrix size 256×186. Twenty four slices of rat brain images with 1 mm slice thickness and no slice gap were collected with a field of view of 100×100 mm in ∼ 2 min acquisition time. Parallel imaging technology at acceleration factor = 2 was used to reduce scan time. A 5-mm diameter reference tube containing 1 ml saline was placed next to the animal for signal intensity (SI) referencing.

Initial MRI scans were performed 8 days after tumor implantation, and rats were randomized into treatment groups so that each group had a similar average tumor size. Rats were treated with saline, Bac-ELP1, or Bac-ELP1-H1 at 200 mg/kg with or without tumor hyperthermia. Treatments began on day 9 and were given daily for four consecutive days. Tumor volume was monitored on days 10, 15, 18, and 22. The MR images obtained from the scanner were transferred to i-site PACS (Philips) for tumor volume analysis. Regions of interest (ROI) were manually traced around the tumor boundaries on every slice that showed signal enhancement indicating tumor presence by an independent radiologist. Tumor volume (mm^3^) was calculated by summing the area of the ROIs around the tumor for all the slices in each rat (control and treated), since the slice thickness was 1 mm.

### Physical Examination for Tumor-induced Neurological Deficits

In order to determine whether treatment with the test agents delayed the onset of neurological deficits and/or prolonged animal survival, rats bearing intracerebral C6 tumors were monitored for the development of neurological deficits during the tumor reduction study described above. A blinded neurological evaluation was performed on animals from each treatment group along with controls using the methods described by Bederson *et al.* [26] with modifications. Briefly, animals were evaluated and assigned a neurological grade score based on the following criteria: (0) no deficit (normal); (1) mild deficit, (2) mild-moderate deficit, (3) moderate deficit, (4) moderate-severe deficit, and (5) severe deficit. Rats were scored in the areas of hemiplegia, gate, grip strength, and ability to grasp an incline plane. Scores in each area were summed for each animal, and the mean of the cumulative scores is reported for each group. A cumulative score of 15 or greater was cause for removal from the study and euthanasia. The date of early removal was recorded for both treated and control animals and used to generate a survival curve.

### Statistical Analysis

Acute biodistribution data and tumor reduction data were analyzed using a one-way ANOVA and a post-hoc Bonferroni multiple comparison. The difference between heated and unheated cell proliferation *in vitro* and the difference between heated and unheated tumor fluorescence in the IVIS experiment was compared using a Student’s t-test. Survival data were analyzed using a Kaplan-Meier analysis with a log-rank test (non-parametric test) of equality of survival functions across groups. A Cox proportional hazard regression models (semi-parametric model) with Breslow method for ties was used to investigate the differences among the groups. Statistics were calculated using Analyze-It for Microsoft Excel or STATA.

## Results

### Effect of Cell Penetrating Peptides on ELP Pharmacokinetics, Tumor Uptake, and Biodistribution

**Figure 1:**
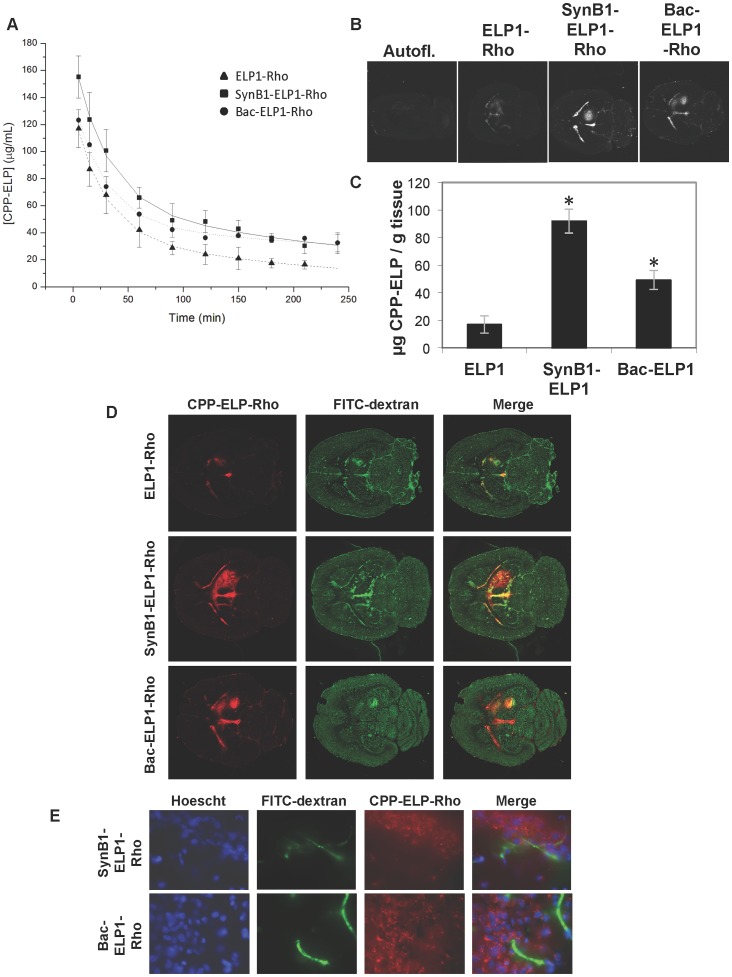
Plasma Clearance and Tumor Uptake of CPP-ELPs. A. Plasma levels with time following IV injection of ELP1-Rho, SynB1-ELP1-Rho, or Bac-ELP1-Rho. Data represent the mean ± s.d. of 6 animals per group. **B**. Representative images of brain sections 4 h after IV injection of rhodamine-labeled polypeptides. **C.** Tumor levels 4 h after IV administration of rhodamine-labeled ELP or CPP-ELPs. Bars, s.e. *, Tumor levels are significantly enhanced (p<0.01, one way ANOVA with post hoc Bonferroni, n = 6 rats/group). **D.** Distribution of rhodamine-labeled polypeptides in tumor and normal brain relative to perfused vasculature. Rhodamine fluorescence was used to follow the localization of the polypeptide within the tumor (left panel), and the perfused vasculature was marked by infusion of high molecular weight dextran 1 min prior to euthanasia (middle panel). **E.** Microscopic images of tumor sections were collected after staining cell nuclei with Hoechst 33342 using a 60× magnification objective.

The effect of CPPs on the pharmacokinetics, tumor uptake, and biodistribution of the ELP carrier was determined using fluorescently labeled polypeptides and quantitative fluorescence analysis. These data were used to determine if the addition of a CPP increased the deposition of the ELP polypeptide in the brain tumor, and the polypeptides’ biodistribution in normal tissues were defined. The SynB1 [27] and Bac [28] CPPs were analyzed since we have previously demonstrated that SynB1 targets the ELP polypeptide to the cell cytoplasm [29] and Bac targets ELP to the cell nucleus [30]. The polypeptides were labeled with Rhodamine (Rho) and administered intravenously to rats bearing intracerebral C6 tumors to determine the pharmacokinetics and biodistributioin of each polypeptide. As shown in Figure 1A, all polypeptides exhibited an initially rapid equilibration out of the blood, followed by a slow clearance. The clearance behavior of all polypeptides was well described by a two-compartment pharmacokinetic model. The pharmacokinetic parameters derived from the data fitting are shown in Table 1. The AUC was largest for Bac-ELP-Rho (33,336.4±6,503.4 µg·min·mL^−1^), followed by SynB1-ELP-Rho (26,985.5±4,092.4 µg·min·mL^−1^), and smallest for unmodified ELP-Rho (13,794.1±3,040.1 µg·min·mL^−1^). Also, Bac-ELP-Rho had the longest terminal plasma half-life at nearly 8 hours, followed by SynB1-ELP-Rho and ELP-Rho at just over 4 hours each.

**Table 1:** Plasma Pharmacokinetics of Each CPP-ELP^a^.

			ELP1	SynB1-ELP1	Bac-ELP1
Initial Concentration	*C_o_*	(ug/mL)	131.8±20.1	170.0±18.6	144.3±12.4
Central Compartment Volume of Distribution	*V_c_*	(mL)	180.6±19.2	189.0±29.9	180.4±18.5
Plasma Clearance	*Cl*	(mL·min^−1^)	1.77±0.4	1.19±0.19	0.80±0.20
Area Under Curve	*AUC*	(µg·min·mL^−1^)	13,794.1±3,040.1	26,985.5±4,092.4	33,336.4±6,503.4
Tissue to Plasma rate constant	*k_tp_*	(min^−1^)	0.014±0.007	0.012±0.003	0.013±0.003
Plasma to Tissue rate constant	*k_pt_*	(min^−1^)	0.021±0.003	0.014±0.004	0.022±0.009
Elimination rate constant	*k_el_*	(min^−1^)	0.010±0.002	0.006±0.0002	0.004±0.0009
Distribution Half Life	*t_1/2,dist_*	(min)	17.6±4.2	23.5±5.1	19.4±5.8
Terminal Half Life	*t_1/2,term_*	(min)	243.4±104.6	277.8±3.9	468.8±85.2

a Plasma clearance data following IV injection of rhodamine-labeled ELP1, SynB1-ELP1, or Bac-ELP1 were fit to a two compartment pharmacokinetic model.

To determine tumor uptake and biodistribution, 4 h after the polypeptide injection, FITC-dextran was injected to mark the perfused vasculature, and brains and major organs were removed, rapidly frozen, sectioned using a cryomicrotome, and analyzed using quantitative fluorescence analysis. Figure 1B shows raw fluorescence images of brains from each treatment group scanned using identical scan parameters. All polypeptides could be detected in the tumor relative to autofluorescence, and polypeptide levels were also high in the CSF, as evidenced by the bright staining of the ventricles. Figure 1B also demonstrates that the SynB1- and Bac-delivered ELP accumulated in tumors at much higher levels than did the non-CPP containing ELP control polypeptide. When these images were quantified relative to the standard curves for each polypeptide, we found that the tumor levels were increased by a factor of 2.9 for Bac-ELP-Rho and 5.4 for SynB1-ELP-Rho relative to unmodified ELP-Rho (p = 0.0001, one way ANOVA, Figure 1C). A similar enhancement in tumor levels by SynB1 and Bac were seen when the tumor levels were compared to plasma levels at either 5 min (C_0_) or 4 h (C_4h_) after injection (Figure S2 A and B). Polypeptide levels were also examined in normal brain tissue and in the ventricles. As shown in Figure S2C, normal brain levels were not significantly affected by the addition of a CPP to ELP, but the SynB1 CPP significantly enhanced polypeptide levels in the CSF as evidenced by fluorescence within the ventricles. When tumor levels were compared to normal brain within the same slices, ELP accumulated in tumors at a 5 fold higher level than in normal brain, and SynB1-ELP and Bac-ELP accumulated in tumors at levels more than 10 fold higher than in normal brain (Figure S2D). Similarly, ELP tumor levels were about 20% of CSF levels, whereas SynB1-ELP and Bac-ELP tumor levels were near 50% of CSF levels (Figure S2E). The high molecular weight FITC-dextran injection demonstrated that there was an increase in the amount of perfused vasculature in the tumor relative to the adjacent normal brain (Figure 1D), but the SynB1-ELP-Rho and Bac-ELP-Rho polypeptide staining extended far beyond the areas of increased vascularity. Microscopic examination of tumor sections revealed that the CPP-delivered ELP was present not only in the blood vessels, but in the extravascular space and within the cells of the tumor (Figure 1E). These data indicate that the ELP polypeptide did passively accumulate in brain tumors in the rat model, and they demonstrate the use of a CPP to enhance both total tumor levels and deposition throughout the tumor relative to the non-CPP containing control. Both SynB1 and Bac significantly increased tumor deposition of ELP, and SynB1 was the more efficient for tumor targeting.

**Figure 2:**
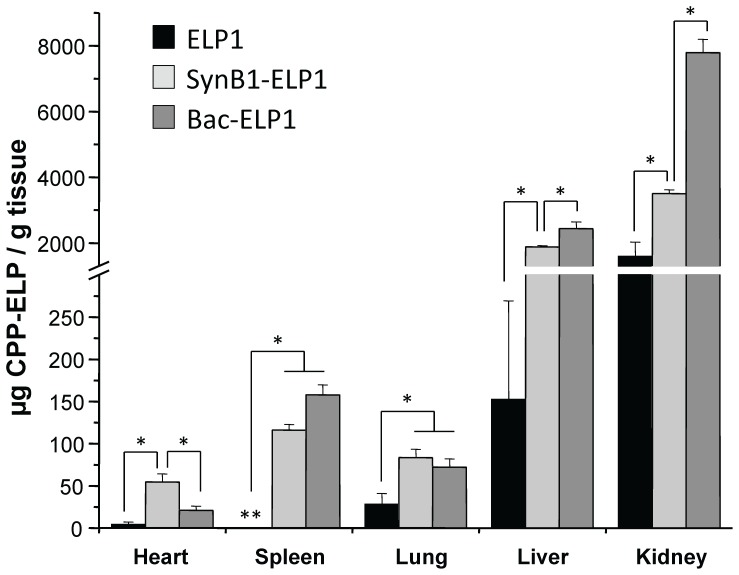
Biodistribution of CPP-ELPs Following IV Injection. Organ distribution of rhodamine-labeled ELP1, SynB1-ELP1, or Bac-ELP1 was determined 4 h after IV administration by quantitative fluorescence analysis. Bars, s.e. *, Organ levels are statistically different (p<0.01, one way ANOVA with post hoc Bonferroni, n = 6 rats/group).

The polypeptide biodistribution in all major organs was also examined 4 h after IV injection using the same quantitative fluorescence analysis. As shown in Figure 2, the use of CPPs not only increased the tumor polypeptide levels, but they also significantly increased polypeptide levels in every organ tested. In the spleen, lung and liver, SynB1-ELP-Rho levels and Bac-ELP-Rho levels were similar, and significantly higher than ELP-Rho levels. Bac-ELP-Rho levels were significantly higher than both SynB1-ELP-Rho and ELP-Rho levels in the kidney, and SynB1-ELP-Rho was the only polypeptide with significantly increased heart levels relative to the other two.

### *In Vitro* Potency of a CPP-ELP Delivered c-Myc Inhibitory Peptide in Glioma Cells

**Figure 3:**
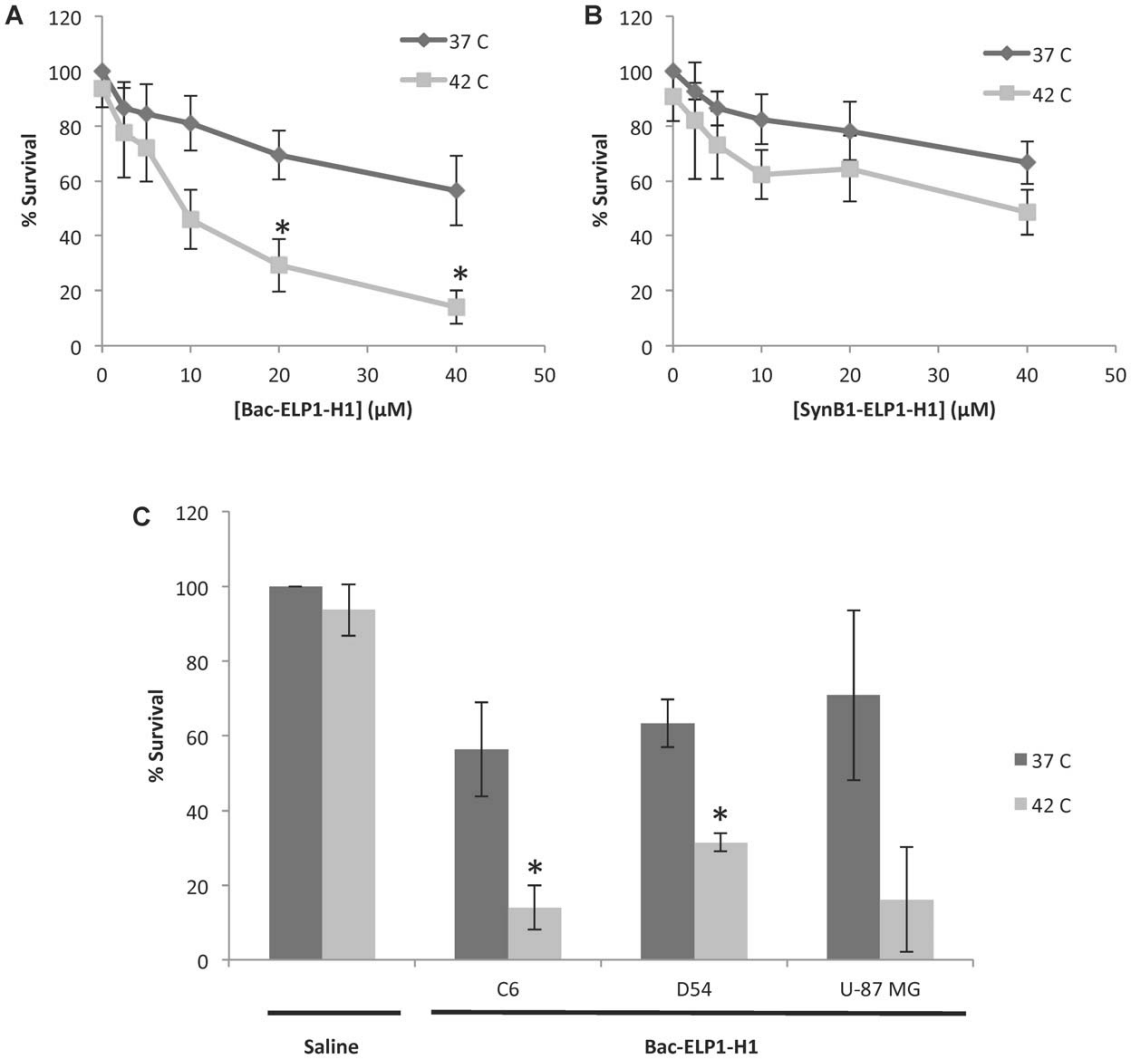
Inhibition of Glioma Cell Proliferation by CPP-ELP-H1 Polypeptides *in vitro*. C6 cells were exposed to various concentrations of Bac-ELP1-H1 (**A**) or SynB1-ELP1-H1 (**B**) at 37°C or 42°C for 1 h, and cell number was determined 72 h later. **C.** C6, D54, or U-87 MG cells were exposed to 40 µM Bac-ELP1-H1 for 1 h at 37°C or 42°C, and cell number was determined 72 h later. Bars, s.e.m. *, Heated and unheated treatments significantly different (p<0.01, Student’s t-test).

Once it was established that ELP could be used to target intracerebral gliomas, its ability to deliver a therapeutic peptide that targets the c-Myc oncogene (called H1) was examined. A previous study determined that the use of the Bac CPP to target the ELP-delivered c-Myc inhibitory peptide (Bac-ELP-H1) to the nucleus was more potent than the use of cytoplasmically targeted CPPs in breast cancer cell lines [30]. Since demonstrating that both Bac and SynB1 enhanced ELP tumor deposition, we assessed the potency of both Bac-ELP-H1 and SynB1-ELP-H1 *in vitro* against several malignant glioma cell lines of human and rat origin, all of which express c-Myc [24], [25], [31]. Both Bac-ELP1-H1 and SynB1-ELP1-H1 inhibited C6 proliferation in a concentration-dependent manner (Figure 3A and B). Also, when aggregation of the polypeptide was induced by incubating the cells at 42°C for 1 h during polypeptide exposure, the antiproliferative effect was enhanced. Under hyperthermia conditions, Bac-ELP1-H1 (9 µM IC_50_) was more potent than SynB1-ELP1-H1 (30 µM IC_50_). These data are consistent with our results in breast cancer cells, where Bac-ELP1-H1 was found to be the most potent inhibitor of cell proliferation, even though it accumulated in cells at lower levels than other CPP-ELP-H1s, due to its ability to enter the cells’ nuclei [30]. The antiproliferative effect of Bac-ELP1-H1 was not limited to C6 cells. As shown in Figure 3C, it also strongly inhibited D54 and U-87 MG human malignant glioma cells, and its potency was enhanced by hyperthermia treatment in all cell lines. Based on the potency and the fact that both Bac and SynB1 significantly enhanced brain tumor uptake, Bac-ELP-H1 was chosen as the lead therapeutic agent for evaluation in efficacy studies *in vivo*.

### Tumor Heating with Infrared Light

**Figure 4:**
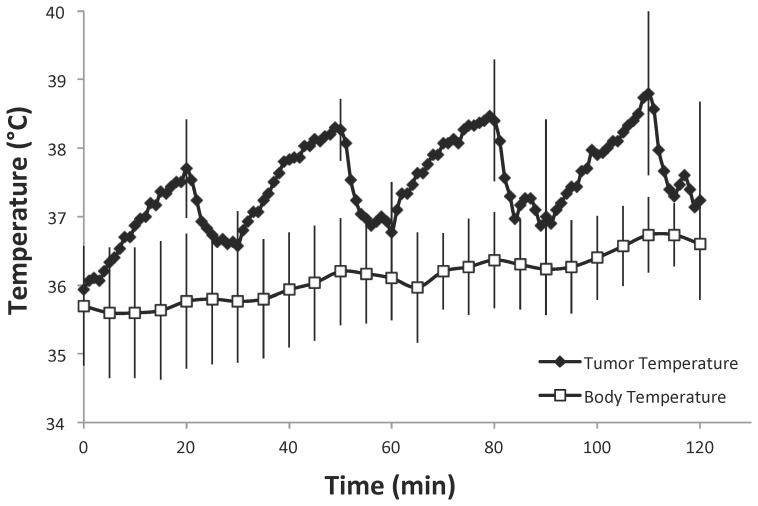
Heating Intracerebral C6 Tumors with Infrared Light. Tumor temperature (as monitored by a needle thermocouple in the tumor core) and body temperature was recorded while illuminating the tumor with 950 nm light from an LED light source. Heating was applied for 20 min, followed by a 10 min cooling period, and this protocol was repeated for 4 cycles. Data represent the mean of three rats, bars, s.d.

Infrared illumination was used in our previous studies as a simple and high-throughput means of delivering hyperthermia to subcutaneous tumors in animal models [13]. In order to determine whether heating intracranial C6 tumors with infrared illumination was feasible, a needle thermocouple was placed into the core of the tumor 10 days after tumor implantation and used to monitor intratumoral temperature while irradiating the tissue with the infrared light source. As shown in Figure 4, activation of the IR light lead to rapid increase in the tissue temperature, and the tissue quickly returned to ≤37°C when the light was turned off. A protocol of cycling between periods of hyperthermia interspersed with periods of normothermia was used because a previous study found this protocol to be superior to long, continuous hyperthermia application for inducing ELP accumulation [32]. Using cycles of 20 min of heating, tumor core temperatures in the desired hyperthermia range of 38–40°C were achieved (maximum tumor temperature of 38.8°C), and the temperature quickly returned to below the polypeptide’s T_t_ during each 10 min cooling period. This target temperature range was chosen because it is sufficient to induce aggregation of Bac-ELP1-H1 in plasma at the dose used ([30] and unpublished data). Due to the sharp temperature transition curve, Bac-ELP1-H1 transitions from completely soluble at 37°C to nearly fully aggregated at 40°C [30]. The rats’ core body temperature was maintained using a homeothermic blanket system, and was not elevated above 37°C during the hyperthermia treatment. These results indicate that the infrared heating system employed here is sufficient for cycling the intracerebral tumor between periods of hyperthermia and normothermia and should allow for very focused activation of polypeptide aggregation and accumulation.

### Thermally Targeted Delivery of Bac-ELP1-H1 to Intracerebral Gliomas

**Figure 5:**
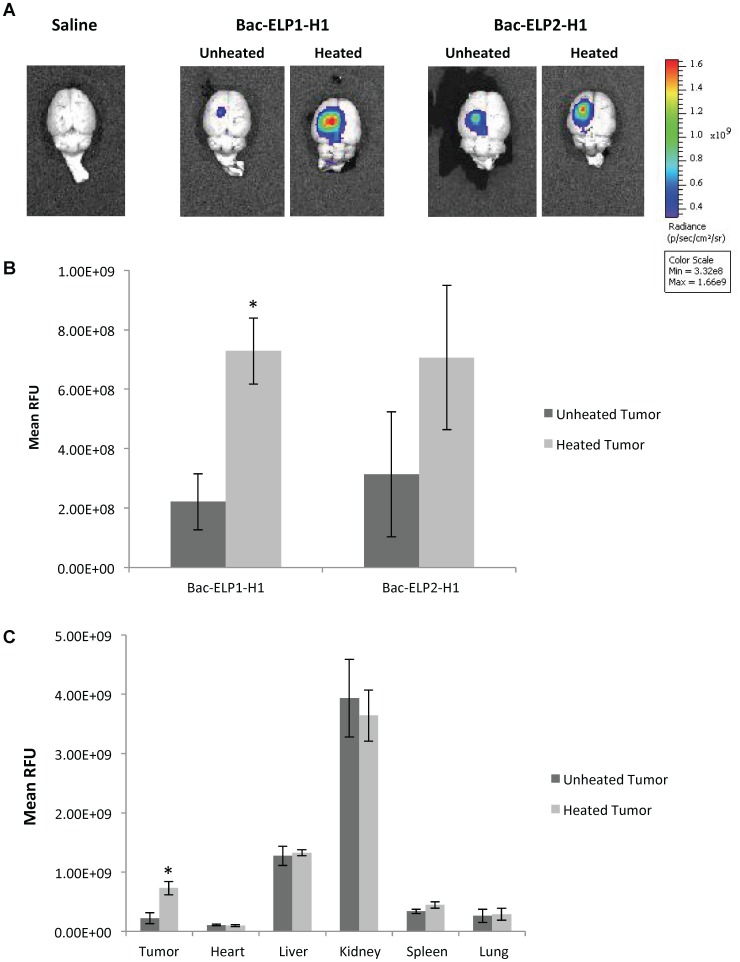
Enhancement of Bac-ELP1-H1 Tumor Uptake by Thermal Targeting. Following IV administration of Alexa750-labeled Bac-ELP1-H1 or Bac-ELP2-H1 with or without hyperthermia, tumor and organ levels were determined by *ex vivo* whole organ fluorescence imaging. **A.** Representative images of brains from each treatment group. **B.** Quantitation of tumor fluorescence from each group. **C.** Quantitation of fluorescence from tumor and all major organs. Bars, s.e.m. *, Fluorescence levels are statistically different (p<0.01, one way ANOVA with post hoc Bonferroni, n = 4 rats/group).

Next, the ability of the Bac-ELP1-H1 polypeptide to be thermally targeted to the tumor site was tested. Rats bearing intracerebral tumors were injected IV with Alexa750-labeled Bac-ELP1-H1, or with an Alexa750-labeled non-thermally responsive control version of the polypeptide, Bac-ELP2-H1. Tumors were heated using the thermal cycling protocol described above, and tumor deposition was determined by *ex vivo* imaging of the rat brains 4 h after the injection using an IVIS Spectrum animal imager. As shown in Figure 5A, the polypeptide naturally accumulated in tumors at a high level relative to the adjacent normal brain. Also, when Bac-ELP1-H1 treatment was combined with tumor hyperthermia, tumor polypeptide levels were noticeably increased. Quantitation of the tumors’ fluorescence intensity revealed that thermal targeting increased Bac-ELP1-H1-Alexa750 tumor accumulation by 3.3 fold (Figure 5B, p = 0.0004, Student’s t-test). In contrast, tumor levels of the non-thermally responsive Bac-ELP2-H1-Alexa750 were increased by only about 2 fold, and the increase was not statistically significant (p = 1.05). These data indicate that a portion of the increased polypeptide levels in the tumor following hyperthermia treatment is due to effects of hyperthermia on the tissue, likely because of increased blood flow to the area. The remainder of the enhanced tumor uptake is due to focused aggregation of the polypeptide at the heated site. Finally, all the major organs were examined by *ex vivo* fluorescence analysis (Figure 5C), and the results were consistent with the data obtained from the quantitative fluorescence analysis of the tissue sections in the prior experiment, suggesting that this method is sufficient to determine biodistribution and indicating that the biodistribution is not significantly altered by addition of the H1 peptide to the Bac-ELP1 carrier. Bac-ELP1-H1-Alexa750 accumulated to the highest levels in the kidney, likely due to clearance of the polypeptide or polypeptide fragments, and also accumulated to high levels in the liver (Figure 5C). Hyperthermia at the tumor site did not affect the polypeptide levels in any other organ.

### Effect of Thermally Targeted Delivery of Bac-ELP-H1 on Tumor Progression, Onset of Neurological Deficits, and Survival

**Figure 6:**
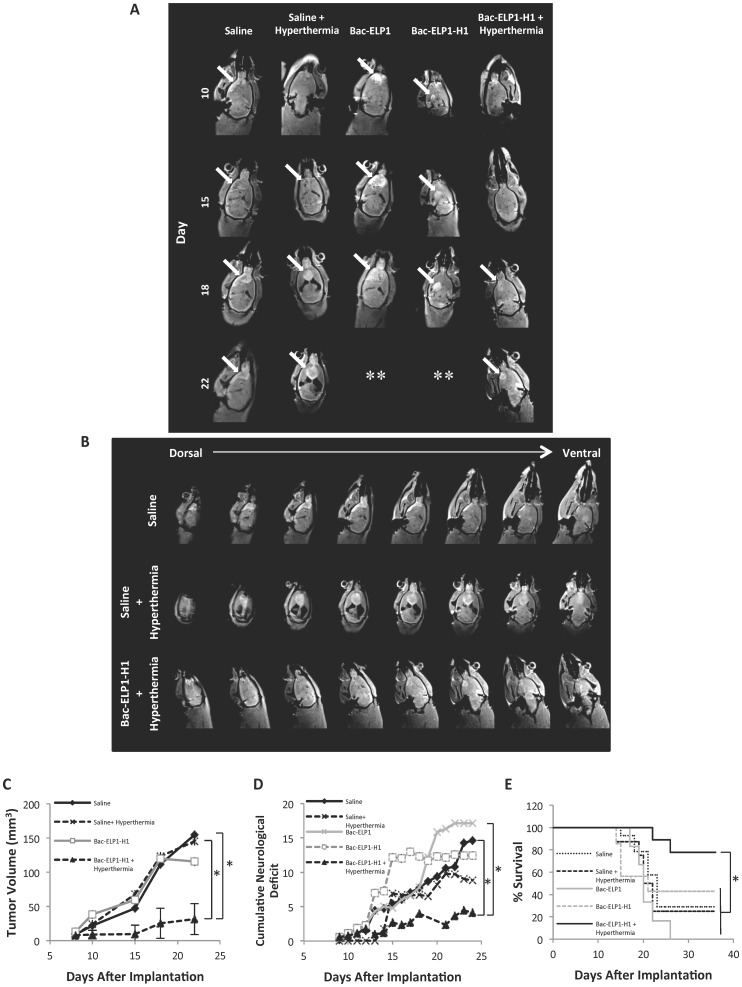
Tumor Reduction, Delayed Neurological Deficits, and Extended Survival by Thermal Targeting of Bac-ELP1-H1. A. Representative MRI images of rats from each treatment group. The arrows point to the tumors’ location. ** Animal didn’t survive to the final scan. **B.** Series of axial sections from a representative animal from saline (top), saline+hyperthermia (middle), and Bac-ELP1-H1+ hyperthermia (bottom) groups. **C.** Tumor volumes derived from MRI scans in each group. **D.** Mean Cumulative Neuroligical Score (on a scale of 0–20, 20 being worst deficit) of each treatment group. Bars, s.e. *, Reductions are statistically significant (p<0.01, one way ANOVA with post hoc Bonferroni, n = 6–9 rats/group). **E.** Kaplan-Meyer survival analysis of rats from each treatment group. *, Survival times are statistically different (p<0.05, log rank test, n = 6–9 rats/group).

Once thermally targeted delivery of Bac-ELP1-H1 to the tumor site was demonstrated, its effects on tumor progression and animal survival were evaluated. Rats bearing intracerebral C6 tumors were treated daily for 4 days beginning on day 9 after implantation. The active Bac-ELP1-H1 polypeptide, or control polypeptides lacking the H1 peptide (Bac-ELP1) or utilizing the non-thermally responsive version of ELP (Bac-ELP2-H1) were injected IV, and, in hyperthermia groups, hyperthermia was applied to the tumor site using the thermal cycling protocol immediately after each injection. Tumor progression was monitored using multi-slice 3D T1 trans-axial imaging with gadolinium-based contrast on days 10, 15, 18, and 22. As shown in Figure 6A, the C6 tumors progressed rapidly in all treatment groups except the Bac-ELP1-H1+ hyperthermia group. By day 22, all control rats had very large tumors present in nearly all brain slices (Top and middle panels, Figure 6B). However, in Bac-ELP1-H1+ hyperthermia treated animals, tumors were small and only visible in one or two slices (bottom panel, Figure 6B). When the tumor volumes were calculated from these images, saline, saline+hyperthermia, and Bac-ELP1 treated tumors reached a total volume of 150 mm^3^. However, in the Bac-ELP1-H1+ hyperthermia group, tumor volumes were 80% smaller, with a mean volume of only 31 mm^3^ (p = 0.004, one way ANOVA, Figure 6C). As the C6 tumors progressed, the rats began to develop noticeable neurological deficits, including hemiplegia, loss of grip strength, and loss of ability to ambulate. These neurological deficits were quantified using a series of assays conducted by an independent observer as described in Material and Methods. Figure 6D shows the results of the cumulative neurological deficit scores for each treatment group. All control groups began to present with deficits around day 14 after implantation. In contrast, the Bac-ELP1-H1+ hyperthermia group showed significant delay in the onset of neurological deficits. By day 24, the deficit severity was reduced by 72% relative to saline treated animals (p = 0.014, one way ANOVA). Once the cumulative neurological score reached ≥15 or rats lost 20% of their pre-treatment body weight, they were removed from the study and euthanized. A Kaplan Meyer analysis of the survival results revealed that there was no significant difference in survival time among the control groups, in which median survival ranged from 20–23 days (Figure 6E). In the Bac-ELP1-H1+ hyperthermia treatment group, survival was significantly increased, with a median survival time of >36 days (p = 0.031, log rank test). In control groups, only 0% to 40% of the rats survived beyond 30 days, as opposed to 80% survival in the Bac-ELP1-H1+ hyperthermia group (Figure 6E). No rats that survived past day 30 died at later time points. A Cox proportional hazard model with Breslow method for ties was used to evaluate group differences. The Bac-ELP1-H1+ hyperthermia treated mice had a 4.63 fold (p = 0.049), 6.12 fold (p = 0.027), and 9.68 fold (p = 0.006) lower hazard rate than saline, saline+hyperthermia, and Bac-ELP1 treated mice, respectively. No overt signs of toxicity or body weight loss during the polypeptide treatment period were seen, and control animals only started to lose weight after day 25 when their neurological deficits were becoming severe (Figure S3). These results demonstrate that thermal targeting of the Bac-ELP1-H1 polypeptide to the brain tumor site resulted in very significant reduction in tumor progression, delayed onset of tumor-induced neurological deficits, and at least doubled survival time relative to control tumors.

## Discussion

In previous studies, we have demonstrated the ability of the ELP polypeptide to deliver various TPs to cancer cells in a thermally targeted manner [9], [15], [29], [30], [33], [34]. We first demonstrated the delivery of the c-Myc inhibitory H1 peptide described here by ELP in breast cancer cells *in vitro* [15], [30], and we recently demonstrated that ELP could be used to thermally target the H1 peptide to breast tumors *in vivo* and significantly inhibit their proliferation [13]. This work extends the application of the ELP-delivered H1 peptide to glioma therapy.

A major hurdle in the treatment of glioma is delivery of drugs to the tumor. We first tested whether the ELP polypeptide would accumulate in brain tumors after IV injection, and whether the addition of cell penetrating peptides to ELP could enhance its uptake in gliomas in a rat model. The ELP polypeptide did accumulate in brain tumors at levels 5 fold higher than in normal brain. This is likely due to the leaky nature of tumor vasculature and the tendency for macromolecules to extravasate and accumulate [35]. Two CPPs, one a basic peptide derived from bovine neutrophils (Bac) [28] and one an amphipathic peptide derived from protegrins (SynB1) [27], were each capable of significantly enhancing brain tumor deposition of the ELP carrier. In addition to enhanced tumor uptake, the CPPs also enhanced the ELP deposition in nearly all the major organs. These results demonstrate that CPPs can be useful for drug delivery applications, but care must be taken in the design of the drug delivery system because the non-specific nature of CPP-based delivery could lead to increased side effects if used for delivery of a highly toxic drug. Little to no toxicity was observed with the Bac-ELP-delivered H1 peptide, and we believe this is a consequence of the peptide’s molecularly targeted nature. Because the peptide targets c-Myc, it will be most potent against tissues that express c-Myc and depend on it for proliferation.

In addition to the use of CPPs to enhance brain tumor uptake, we also demonstrated that ELP could be used to thermally target the H1 peptide to the orthotopic brain tumors using a heat cycling protocol of targeted tumor hyperthermia. This is a significant advancement because it is the first demonstration of thermally targeting ELP to a brain tumor. The heat cycling protocol was utilized because it has been previously shown to be superior to a single long, continuous heating period [32]. The rationale behind this approach is that during the hyperthermia period, the polypeptide aggregates and accumulates in the tumor vasculature. Because ELP aggregation is fully reversible, when the tissue is cooled below the transition temperature, the polypeptide will re-dissolve. This creates a concentration gradient from the intravascular space to the extravascular space down which the polypeptide diffuses. Multiple rounds of heat cycling allow more polypeptide aggregates to be trapped in the tumor vasculature with each heat cycle, and ultimately this polypeptide re-dissolves and extravasates when the heat is removed [32].

Tumor heating lead to a statistically significant enhancement of Bac-ELP1-H1 levels and an increased but not statistically significant enhancement of Bac-ELP2-H1 levels. Since Bac-ELP2-H1 does not aggregate at the hyperthermia temperature used in this study, this trend toward increased levels likely indicates that tissue heating affects the total perfusion or enhances the vascular permeability. These effects, combined with the thermally triggered aggregation, work together to contribute to the significant enhancement of tumor levels observed with Bac-ELP1-H1.

Not only was the brain tumor uptake enhanced with focused hyperthermia, also thermal targeting of the Bac-ELP1-H1 polypeptide to the tumors resulted in 80% inhibition of tumor volume, significantly delayed onset of neurological deficits, and at least doubled survival. These results demonstrate that use of ELP to thermally target the H1 peptide for glioma therapy is a promising approach. The use of the C6 model allowed us to validate the therapeutic in an aggressive, fully immunocompetent preclinical model that histologically mimics the human disease. However, the limitations of the C6 model are that the tumors are not of human origin, and due to the outbred nature of the original rat strain, the tumors are not syngeneic to any inbred strain and can therefore be immunogenic [36]. Tumor immunogenicity was not a major obstacle in this model given that we had 100% tumor formation from our cell implantations. However, it is possible that tumor immunogenicity played a role in the small number of saline treated animals that survived the study (<30%), and we can not rule out that the animals’ immune system played a role in clearing Bac-ELP-H1 treated tumors. Future studies will expand this testing into other GBM models, including mouse orthotopic xenografts of human glioblastoma cells.

Clinically, this technology could be instituted in several ways. Targeted hyperthermia of even deep-seated tumors in soft tissue is feasible using high intensity focused ultrasound (HIFU) [37], [38], and MRI-guided HIFU provides a mechanism for image guided ultrasound therapy and real-time monitoring of the temperature of the heated tissue [39], [40]. Though normally used for ablation therapy, HIFU can be used to apply the mild hyperthermia conditions needed for ELP thermal targeting by controlling the acoustic power and moving the beam in concentric circle trajectories [41]. In the case of a resectable brain tumor with high c-Myc expression, the Bac-ELP1-H1 polypeptide could be administered following surgery, and the tumor site could be thermally targeted using HIFU before the craniotomy is repaired. Also, research is advancing in the field of transcranial HIFU [42], which alleviates the need for a craniotomy. This technology could allow repeated, non-invasive treatments after surgery to prevent or treat recurrence, and it could allow this therapy to be applied to treat inoperable tumors.

More broadly, these data demonstrate that, given a suitable carrier, peptide-based therapeutics can be efficacious for cancer therapy. These studies pave the way for expansion of the ELP technology for delivery of other therapeutic peptides, which may be applied for any type of solid tumor therapy. As we learn more about the etiology of various tumor types, we will certainly elucidate promising targets for which we have no small molecule drugs. The use of peptide therapeutics, which may be easily and rationally designed for any given target protein, delivered by the ELP carrier, represents a promising new frontier for developmental cancer therapeutics.

## Supporting Information

Figure S1 **Histology of Intracerebral C6 tumors.** **A.** A whole brain H&E image of a rat brain containing a small C6 tumor was obtained by transillumination and digital photography. 10× and 20× magnified images were collected using a Nikon microscope equipped with a digital camera. **B.** 20× magnified image of a C6 tumor 14 days after implantation. Adjacent sections were stained with H&E or GFAP. Scale bar = 0.1 mm.
(TIFF)

Figure S2 **A and B.** Tumor polypeptide levels relative to plasma polypeptide levels at 5 min after injection (C_0_, A.) or 4 h after injection (C_4h_, B.). **C.** Total levels of each polypeptide in normal brain, tumor, and ventricles. **D.** Ratio of tumor polypeptide levels to normal brain polypeptide levels within the same brain section. **E.** Ratio of tumor polypeptide levels to CSF polypeptide levels in the ventricles. * Levels are statistically significantly increased relative to ELP1 (p<0.01, one way ANOVA with post hoc Bonferroni). ** Levels of Bac-ELP1 in normal brain were below the lowest values of the standard curve.
(TIFF)

Figure S3 **Mean body weight of rats from each treatment group shown in the tumor reduction study in** **Figure 6** **.** Rats were treated with the indicated agents on days 9, 10, 11, and 12.
(TIFF)
