# Supplementary figures and images for: Thermally Targeted Delivery of a c-Myc Inhibitory Polypeptide Inhibits Tumor Progression and Extends Survival in a Rat Glioma Model
Source: PLoS One. 2013 Jan 25;8(1):e55104. doi: 10.1371/journal.pone.0055104 (PMC3555869; doi:10.1371/journal.pone.0055104)

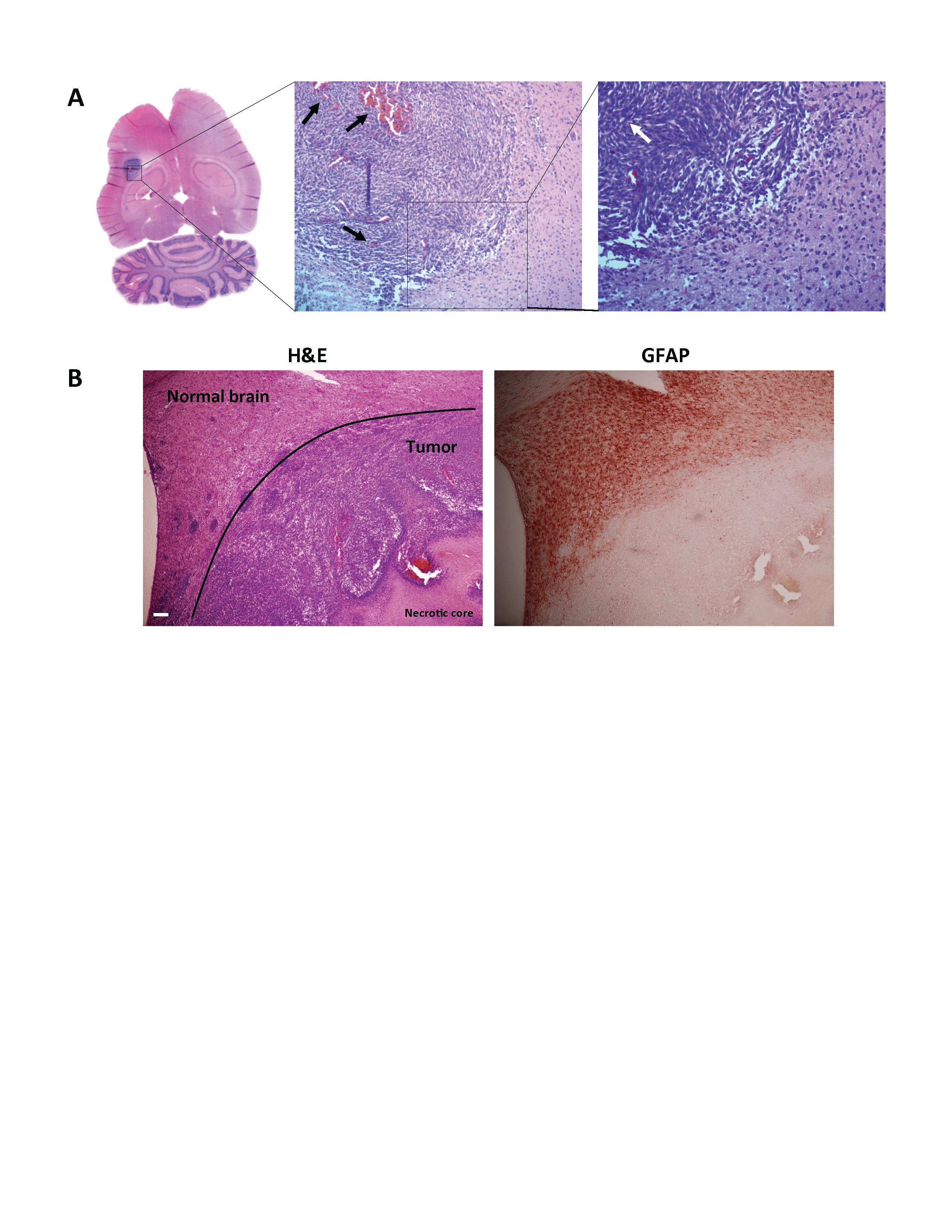

Supplement: Figure S1 — Histology of Intracerebral C6 tumors. A. A whole brain H&E image of a rat brain containing a small C6 tumor was obtained by transillumination and digital photography. 10× and 20× magnified images were collected using a Nikon microscope equipped with a digital camera. B. 20× magnified image of a C6 tumor 14 days after implantation. Adjacent sections were stained with H&E or GFAP. Scale bar = 0.1 mm. (TIFF) [file pone.0055104.s001.tiff]

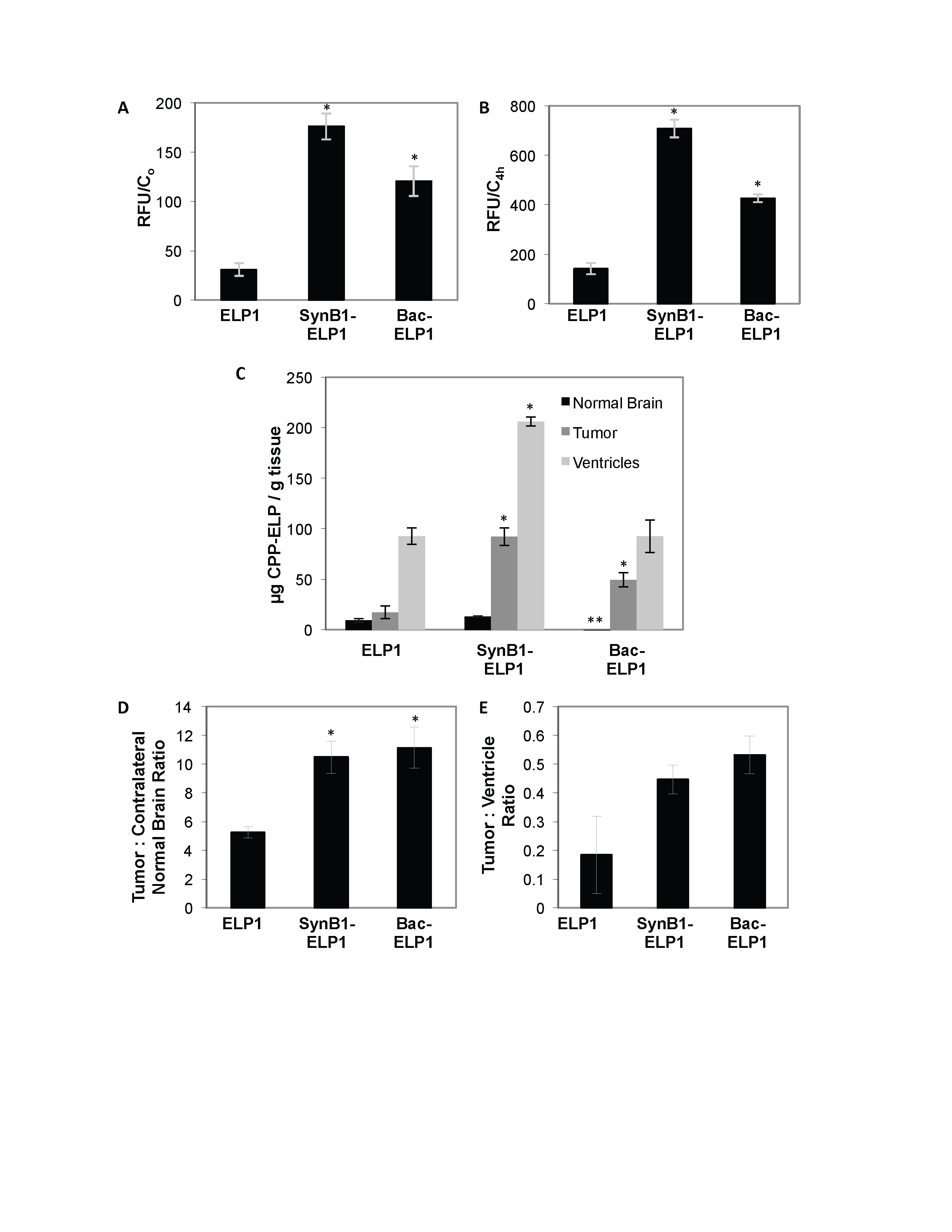

Supplement: Figure S2 — A and B. Tumor polypeptide levels relative to plasma polypeptide levels at 5 min after injection (C0, A.) or 4 h after injection (C4h, B.). C. Total levels of each polypeptide in normal brain, tumor, and ventricles. D. Ratio of tumor polypeptide levels to normal brain polypeptide levels within the same brain section. E. Ratio of tumor polypeptide levels to CSF polypeptide levels in the ventricles. * Levels are statistically significantly increased relative to ELP1 (p<0.01, one way ANOVA with post hoc Bonferroni). ** Levels of Bac-ELP1 in normal brain were below the lowest values of the standard curve. (TIFF) [file pone.0055104.s002.tiff]

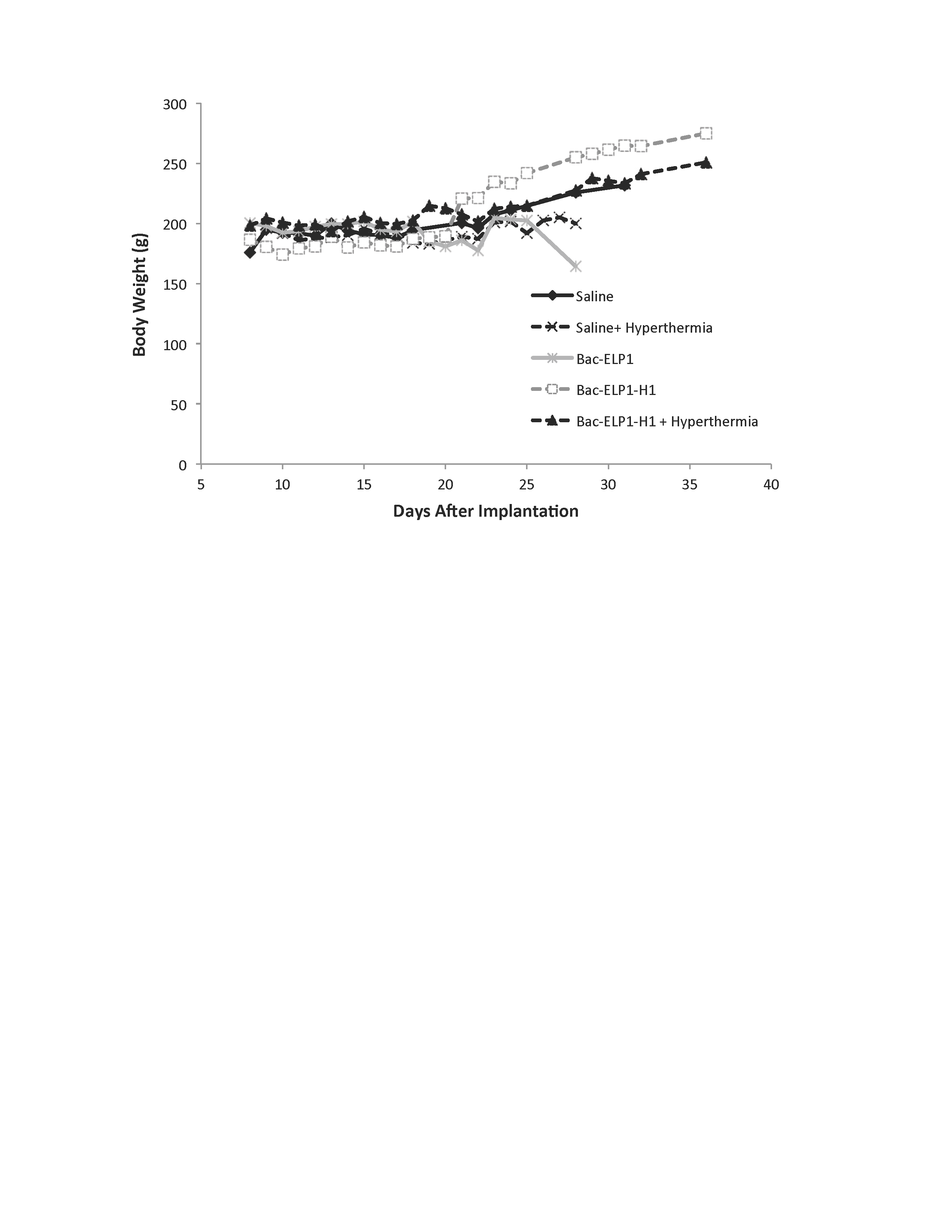

Supplement: Figure S3 — Mean body weight of rats from each treatment group shown in the tumor reduction study in Figure 6 . Rats were treated with the indicated agents on days 9, 10, 11, and 12. (TIFF) [file pone.0055104.s003.tiff]
